# Psychopathy and Economic Behavior Among Prison Inmates: An Experiment

**DOI:** 10.3389/fpsyg.2021.732184

**Published:** 2021-09-20

**Authors:** Loukas Balafoutas, Aurora García-Gallego, Nikolaos Georgantzis, Tarek Jaber-Lopez, Evangelos Mitrokostas

**Affiliations:** ^1^Department of Public Finance, University of Innsbruck, Innsbruck, Austria; ^2^Department of Economics, Universitat Jaume I, Castellón de la Plana, Spain; ^3^Burgundy School of Business-School of Wine & Spirits Business, Dijon, France; ^4^Economix, Université Paris Lumière, Univ Paris Nanterre, Centre National Recherche Scientifique, Nanterre, France; ^5^University of Portsmouth, Portsmouth, United Kingdom

**Keywords:** psychopathy, pro-social behavior, prison inmates, lab-in-the-field, experiment

## Abstract

This paper investigates whether there is a connection between psychopathy and certain manifestations of social and economic behavior, measured in a lab-in-the-field experiment with prison inmates. In order to test this main hypothesis, we let inmates play four games that have often been used to measure prosocial and antisocial behavior in previous experimental economics literature. Specifically, they play a prisoner's dilemma, a trust game, the equality equivalence test that elicits distributional preferences, and a corruption game. Psychopathy is measured by means of the Levenson Self-Report Psychopathy Scale (LSRP) questionnaire, which inmates filled out after having made their decisions in the four games. We find that higher scores in the LSRP are significantly correlated with anti-social behavior in the form of weaker reciprocity, lower cooperation, lower benevolence and more bribe-oriented decisions in the corruption game. In particular, not cooperating and bribe-maximizing decisions are associated with significantly higher LSRP primary and LSRP secondary scores. Not reciprocating is associated with higher LSRP primary and being spiteful with higher LSRP secondary scores.

## Introduction

The World Prison Population List^1^ gives its readers information on the number of prisoners held in the territories of 222 countries worldwide. Although comparability of imprisonment rates across countries must be subject to caution, data show that the overall prison population has been increasing in the last four decades. The United States of America currently hold over 2.3 million people in prison (Sawyer and Wagner, 2020), which represents the highest prison population rate in the world. The costs for correctional spending and crime combat are the fastest growing budget item after Medicaid (Henrichson and Delaney, 2012). Calculating the costs of criminal activity is quite difficult, since they vary widely among various offense categories. For instance, estimates place the total cost of crime in England and Wales at £60 billion in the year 2000 (Brand and Price, 2020). The vast amount of costs generated by criminal incidents and the attempt to administrate its consequences make it necessary to better understand the underlying nature of criminal behavior.

Anti-social and criminal behavior can partly be explained by various personality disorders. One of them is psychopathy, a personality disorder defined by a lack of empathy for others. This disorder is related to antisocial disposition and characterized by having impaired empathy or lack of remorse, egotistical personality traits and sometimes even expressing cold blooded behavior toward others. Brandt et al. (1997) estimate that the base rate of psychopathy among prison population is as high as 37%. This high prevalence of psychopathic traits means that examining the relationship between such traits and behavior among criminals is of particular value.

The objective of this study is to investigate whether there is a connection between psychopathy and certain manifestations of social and economic behavior, measured in a lab-in-the-field experiment with inmates. To the best of our knowledge, this is the first study connecting psychopathic traits with social and economic behavior in a prison environment. Hence, our main research question is: Can psychopathic traits explain social and economic behavior among inmates? Many dilemmas in economic situations involve a conflict between selfish monetary reward maximization and devotion to ethical, pro-social norms connected to inferior economic benefit. The existence of ethical behavioral patterns among institutionalized subjects is of great interest as the starting point of rehabilitation and social inclusion strategies based on the principle that everyone is ethical to some extent.

To answer our research question, we use four games that have often been used to measure prosocial and antisocial behavior in the experimental economics literature: a prisoner's dilemma (henceforth PD), a trust game (henceforth TG), the equality equivalence test that elicits distributional preferences (henceforth EET), and a corruption game (henceforth CG). This choice of games is motivated by the fact that trust, reciprocity, cooperativeness, and distributional preferences are behavioral traits of essential importance for a successful rehabilitation of inmates into social and professional life after their release from prison (see Balafoutas et al., 2020, for a discussion). In addition, our study is the first to collect data on inmates' actions in a game meant to capture essential aspects of a corruption setting. Data from the corruption game allow us to study inmates' decisions when facing a social dilemma that includes an ethical component. We correlate behavior in all these games to a measure of psychopathy based on the Levenson Self-Report Psychopathy Scale (henceforth LSRP).

The data collection took place as part of a lab-in-the-field experiment conducted with 176 inmates in two prisons in Chania, Greece. Inmates played the games described above, in a number of sessions conducted within prison and following standard experimental protocol (regarding randomization, anonymity, and the use of monetary incentives). It is important to note that not all inmates played the four games. Out of the 176 inmates, 71 were recruited in the 2015 sessions and decided only on the CG, and 105 were recruited in 2016–2017 and decided on the TG, PD, and EET. The behavioral data from the economic games are complemented by administrative and survey data, including the LSRP.

Our results reveal that psychopathy as measured in the LSRP explains several aspects of inmates' social behavior. Higher scores in the LSRP are significantly correlated with anti-social behavior in the form of weaker reciprocity, lower cooperation, lower benevolence (implying a higher likelihood that a person is classified as having spiteful distributional preferences), and more bribe-oriented decisions in the corruption game.

## Literature Review

### Economic Experiments in Prisons

Despite the large economic and social costs of crime and the importance attributed by society and policymakers on the rehabilitation of criminal offenders, there is a relative scarcity of economic research on the behavior of prison inmates. Recently, a few studies using experimental economic methods have successfully overcome the practical and administrative challenges linked to this kind of research, yielding valuable insights on several aspects of the social and economic behavior of prison populations. One lesson that can be drawn from this literature is that differences in pro-social behavior (mainly altruism and cooperativeness) between prison inmates and samples of non-criminals are not systematic or consistent. Some studies find either very small, or negligible differences (Birkeland et al., 2014; Chmura et al., 2016), while others suggest that prison inmates are less pro-social than other groups of participants (Clark et al., 2015), or even more pro-social in some cases (Khadjavi and Lange, 2013; Nese et al., 2016).

Besides documenting patterns of behavior among inmates and comparing them to different samples, a few recent studies in prisons have considered topics such as the deterrence effect of punishment on antisocial behavior (Khadjavi, 2015), criminal identity and ethical behavior (Cohn et al., 2015), and the existence of in-group bias within a stigmatized group such as prison inmates (Balafoutas et al., 2020). Guo et al. (2020) differentiate between inmates' behavior toward an in-prison and out-of-prison sample and show that a simple priming intervention can promote rehabilitation by strengthening inmates' pro-social behavior toward the out-group. The current study uses, in part, the same sample as Balafoutas et al. (2020), but it studies an entirely different and hitherto unanswered question on the relationship between psychopathy and behavior among prisoners.

### Psychopathy and Economic Behavior

Cleckley (1956) defines psychopathy as being manipulative, egocentric, impulsive, deceitful, and exhibiting antisocial behavior. The partial overlap between this definition and the purely self-interest notion of *homo oeconomicus* initiated a series of studies investigating whether psychopathy or psychopathic personality traits are linked to entrepreneurial abilities and success. Babiak et al. (2010) estimated that the general psychopathy prevalence is three times higher among the business workforce compared to the general population. Akhtar et al. (2013) argued that a certain degree of manipulativeness and callousness, both psychopathic characteristics, can be necessary for high achievements in a respective business field. Walters (2004) remarks that the primary psychopathic personality traits such as “superficial charm, deceit, lack of guilt read like the job description of a good car salesman or a politician” (page 144). Akhtar et al. (2013) report moderate correlation between entrepreneurial activities and psychopathy but provide only weak support for the stereotype of a “corporate psychopath.” They find that primary psychopathy is negatively correlated to “social entrepreneurship,” i.e., initiate social activities such as improving the community, enhance education, or create student organizations. Similar conclusions have been obtained by Hassall et al. (2015) who measure academic success and psychopathic personality traits among business and psychology students and find that business students score significantly higher on psychopathy scores—albeit without a significant effect on academic success. One lesson that emerges from this strand of the literature is that we need to better understand psychopathic traits.

The literature in experimental economics that relates psychopathy to behavior in economic games is rather scarce, and at the same time highly relevant for our work. In a lab experiment, Ibáñez et al. (2016) study the relationship between emotions and trust. As a sign of the manipulative stage of a psychopath's behavior, they find that higher psychopathy scores are correlated with non-reciprocal decisions. A similar branch of the literature has examined the relationship between psychopathy and cooperative behavior in economic games. Mokros et al. (2008) find that psychopaths in a high-security psychiatric hospital behave in a non-cooperative manner in a prisoner's dilemma. Montañes et al. (2003) use various modifications of the prisoner's dilemma and show that Antisocial Personality Disorder correlates with non-cooperative behavior. Rilling et al. (2007) use a sample of 30 non-clinical subjects whose psychopathy is assessed using LSRP scores and Psychopathic Personality Inventory (PPI). In the repeated version of the prisoner's dilemma, they find a high correlation between non-cooperative behavior and higher LSRP scores among the male participants of their sample. On the contrary, they find no effect of psychopathy measured by the PPI. Curry et al. (2011) report that individuals with higher scores in the Machiavellian Egocentricity subscale of the PPI are less likely to behave cooperatively. Hence, considering cooperative behavior as a metric of empathy and a pro-social inclination, research so far indicates that psychopathy relates negatively to cooperation in social dilemma situations^2^^,^^3^. Our paper contributes to this branch of the literature by being the first to examine the relationship between psychopathy and various measures of prosocial behavior in a sample of imprisoned subjects with a verified criminal record.

### Psychopathy and Criminal Behavior

The definition of psychopathy by Cleckley (1956) suggests that a number of negatively perceived personality traits should be considered core characteristics of psychopaths. On the other hand, observing antisocial behavior clearly is not sufficient to categorize someone as a psychopath. Most prison inmates, for instance, would be considered as antisocial to a certain degree, while only a minority of them expresses psychopathic personality disorders (Levenson et al., 1995). Nevertheless, antisocial behavior, impulsivity, lack of remorse, and the proneness toward violence is often seen as an explanation for why psychopaths tend to show more aggressive behavior among the institutionalized population. Vaughn et al. (2009) examine the potential subtypes of psychopathy among incarcerated juveniles and find that offenders scoring high in psychopathic measures indicate a greater likelihood of participating in self and other-destructive behavior than non-psychopathic juveniles. Compared to other criminals, psychopaths commit a significantly higher number of crimes and more violent ones (Hare and McPherson, 1984). Although some researchers have opposed these findings and conceded psychopathy only a limited role in crime forecasting, they nevertheless acknowledge the need for further research into psychopathic personality traits and the behavior of criminals (Walters, 2004).

Forecasting the likelihood of criminal acts would be without doubt a useful tool for police resource allocation and a potential way to reduce costs caused by the imprisoned population. Hence, improving crime prediction using models based on regularity and space clusters combined with psychological risk assessments that predict antisocial behavior should be considered (Brinkley et al., 2008; Johnson, 2010). Models developed for crime forecasts presently concentrate on social status, locality and crime opportunity, but individual characteristics are growing in importance, especially since crimes committed in affect are hard to account for (Miller et al., 2008; Johnson, 2010). Current research appears to regard psychopathy as a promising indicator for violence even among the female population (Levenson et al., 1995)^4^.

It is worth noting that Miller et al. (2008) and other researchers (Porter et al., 2001; Skeem et al., 2007) assume that primary psychopaths are born with such a predisposition, whereas secondary psychopaths are believed to be shaped by their environment. The first to introduce such a distinction was Karpman (1948) who proposed a re-orientation of the concept of psychopathic personality. Specifically, he suggested to divide it into two main groups: the symptomatic or secondary psychopathy, and the primary, essential, or idiopathic psychopathy. Under the heading of secondary psychopathy are included the psychoses and neuroses that have a strong antisocial or delinquent aspect. Individuals of the other, primary group, suffer from a disease of its own designated as anethopathy. This is a mental disease, characterized by a personality organization having in particular a virtual absence of any redeeming social reaction (conscience, guilt, binding and generous emotions, etc.), while purely egoistic, uninhibited instinctive trends are predominant. These are as close to the constitutional as can be found. Despite the clarity of past literature, there are still many empirical studies that investigate the frontier between primary and secondary psychopathy^5^. Further research will shape the view of psychopathy as either being an inborn or a molded personality disorder.

### Measuring Psychopathy

There exists a diverse selection of measurement tools to assess psychopathy. Two popular ones are the Hare Psychopathy Checklist-Revised (PCL-R) also sometimes called Psychopathy Checklist—revised (Hare and Neumann, 2006) and the Levenson Self-Report Psychopathy Scale (Levenson et al., 1995). The PCL-R is constructed as a semi-structured interview and additionally uses official records (Hare and Neumann, 2006). It is capable of addressing both primary and secondary, psychopathic subgroups (Hare and Neumann, 2006). From a cost effectiveness perspective, the PCL-R has some notable disadvantages. For example, it is necessary that a trained clinical expert executes the interview, which is relatively time consuming (Lynam et al., 1999; Brinkley et al., 2001). Moreover, its development is primarily based on male offenders and requires historical records (Lynam et al., 1999; Brinkley et al., 2001).

Based on Karpman's (1948) initial distinction, Levenson et al. (1995) studied antisocial dispositions among non-institutionalized populations and developed the well-known Levenson Self-Report Psychopathy scale (LSRP). The LSRP is a self-report measure which was designed to assess primary and secondary psychopathic features in non-institutionalized populations. It is an advantage that it does not require historical crime records. Lynam et al. (1999) regard, based on their findings, the LSRP-Scale as a reasonable measure for psychopathy in context of variant measurements. Miller et al. (2008) conclude that the LSRP is significantly related to personality traits commonly seen in psychopathic individuals such as agreeableness and narcissistic behavior. Furthermore, the LSRP is strongly correlated with negative emotionality and other personality disorder symptoms.

Hence, both self-report tests (PCL-R as the LSRP) are capable of measuring psychopathic tendencies reliably to various degrees (Zolondek et al., 2006; Brinkley et al., 2008; Miller et al., 2008; Becker et al., 2012). Given that the LSRP is less time consuming and does not require historical crime records, we selected it for the present study.

## Experimental Design

Our experimental design is based on four simple economic games^6^ and the Levenson Self-Report Psychopathy (LSRP) scale, supplemented by a collection of socio-demographic data, questions related to inmates' experience inside the prison and data provided by the prison administration.

### The Games

#### Trust Game

We use a discrete version of the trust game (Berg et al., 1995). Subjects are matched in groups of two and are randomly assigned one of two roles in a between-subjects design: player 1 (sender), or player 2 (receiver). The sender has two strategies, to trust or not to trust the receiver. If he does not trust, both players earn an outside option of €10 each. If he trusts, the total available surplus is doubled (€40) and the receiver is then asked to take one of two actions: she can either reciprocate the sender's trust by implementing an equal split of €20 for each player, or choose the non-reciprocal action and keep €35 for herself, leaving the sender with only €5^7^. While trust and reciprocity lead to an improvement and a doubling of payoffs for both players, the subgame perfect equilibrium prediction for this game is that receivers never reciprocate trust, and anticipating this, senders never trust^8^.

#### Prisoner's Dilemma

We use the same version of the simultaneous prisoner's dilemma as Balafoutas et al. (2020) and Khadjavi and Lange (2013), depicted in Table 1. Two players simultaneously decide either to cooperate with the other player or to defect. The dominant strategy for both players—and hence the Nash equilibrium—is defection, while choosing to cooperate is the pro-social action that leads to a Pareto improvement in payoffs if it is chosen by both players.

**Table 1:** The prisoner's dilemma.

		**Player 2**	
		**Defect**	**Cooperate**
Player 1	Defect	3, 3	9, 1
	Cooperate	1, 9	7, 7

#### Equality Equivalence Test

In contrast to all other games, the Equality Equivalence Test (Kerschbamer, 2015) entails no strategic interaction. This test elicits distributional preference types by asking each subject to make ten binary choices between an equal and an unequal allocation, involving an own payoff and a payoff for a randomly matched subject. The ten choices are shown in Table 2, broken down into a disadvantageous inequality block and an advantageous inequality block, referring to the direction of inequality as seen from the perspective of the decision maker. The ten choices, and in particular the row at which the subject switches from the equal to the unequal allocation, allow us to classify all subjects into one of four basic distributional preference types: altruistic (or efficiency loving), inequality averse, spiteful, and inequality loving^9^.

**Table 2:** The equality equivalence test (EET).

**Left**				**Right**	
**You**	**Another**			**You**	**Another**
	**person gets**				**person gets**
**Disadvantageous Inequality Block**					
3.2	5.2	LEFT	RIGHT	4	4
3.6	5.2	LEFT	RIGHT	4	4
4	5.2	LEFT	RIGHT	4	4
4.4	5.2	LEFT	RIGHT	4	4
4.8	5.2	LEFT	RIGHT	4	4
**Advantageous inequality block**					
3.2	2.8	LEFT	RIGHT	4	4
3.6	2.8	LEFT	RIGHT	4	4
4	2.8	LEFT	RIGHT	4	4
4.4	2.8	LEFT	RIGHT	4	4
4.8	2.8	LEFT	RIGHT	4	4

#### CG

The Corruption Game (CG) framework studied here is based on Jaber-López et al. (2014). In a framed interaction protocol, two subjects in the role of “firms” bid in quality (*Q*) and bribe (*B*) levels (both in integers ranging between 0 and 10, *Q* + *B* = *10*), for the procurement of a “public project,” the quality of which is beneficial to all players within a group and individually profitable to the winning firm. A third subject in the role of a “public official” chooses the winning proposal having full information on the two firms' bids. Payoffs in the CG are determined as follows:


$$\begin{array}{l} \Pi_{official}=10+\frac{1}{2}Q_{winner}+B_{winner}\\ \Pi_{winner}=10+\frac{1}{2}Q_{winner}-2B_{winner}+10\\ \Pi_{loser}=10+\frac{1}{2}Q_{winner} \end{array}$$

Assuming rational and selfish subjects, there are three pure strategy Nash equilibria for firms' behavior with a discrete strategy space in this game: (*Q, B*) = (7, 3), (*Q, B*) = (6, 4), and (*Q, B*) = (5, 5). Rational and selfish public officials maximize earnings therefore the subgame perfect Nash equilibrium predicts that they will choose the firm that offers the highest bribe. This framework represents a social dilemma, in the form of a tradeoff between quality and bribes. For firms, higher bribe payments indicate lower pro-sociality, since they imply sacrificing social welfare in the interest of increasing one's likelihood of winning the prize. For public officials, bribe-maximizing (as opposed to quality-maximizing) choices capture selfish, anti-social behavior, while officials driven by pro-social motives may sacrifice part of their own monetary earnings in favor of a higher quality project.

### Levenson Self-Report Psychopathy Scale and Questionnaires

As already mention in section Measuring psychopathy, the evaluation of psychopathy in our paper is based on the LSRP (Levenson et al., 1995). Respondents state their degree of agreement with each of 26 statements, on a Likert-scale ranging from 1 (“totally disagree”) to 4 (“totally agree”)^10^. One attractive feature of this scale is that it elicits the level of psychopathic elements in a respondent's personality by offering three types of information, namely an aggregate measure of psychopathy (comprising all 26 questions) and two specific ones: *primary*, which refers to selfishness, lack of caring, manipulation of others and callous attitudes and is based on the first 16 questions; and the *secondary* psychopathy scale, associated with an impulsive, volatile or self-destructive personal style and is based on the last 10 questions (see Levenson et al., 1995; Lynam et al., 1999). All questions are shown in Supplementary Material 3.

Psychopathy by definition comprises manipulative and abusive behavior. In particular, individuals who display psychopathic traits are considered to be able to manipulate others in order to achieve personal benefits, without guilt or unfairness entering their moral considerations. Therefore, in our experiment, we expect higher scores on the psychopathy scale to be associated with less cooperative and pro-social behavior.

Inmates were asked to fill out the LSRP questionnaire after having made their decisions in the four games (TG, PD, EET, and CG). They were also asked to provide socio-demographic information (on their nationality, age, marital status, education level, and number of siblings). Additionally, we asked them to answer some questions regarding the conditions of their imprisonment: time spent in the current prison, number of times imprisoned, total time spent in prison during their life, type and length of sentence, attendance of religious activities in prison, number of cell mates, frequency of leaving the prison (for any reason) and number of working days per month. The prison administration provided us with this same information, allowing us to double check and correct for minor discrepancies.

### Procedures

In January 2015 we ran one session in the low security agricultural prison facility of “Agia” and one session in the high security prison facility “Crete 1,” in which subjects played only the CG. In November 2016 we ran one session in the high security prison and two simultaneous sessions in the low security agricultural prison, in which subjects played the PD, TG, and EET. In April 2017 we conducted an additional session in the low security prison and again subjects played PD, TG, and EET. In all sessions, subjects also filled out the LSRP questionnaire. We recruited volunteer male inmates by posting announcements around the prison premises. Additionally, 2 days before each session, the experimenters went to the prison to answer possible questions and give a short explanation of what is an economic experiment. Once they decided to participate, inmates had to register through the prison administration.

All sessions took place either in the prison's gym or in the library. No guards were present and we insisted on and guaranteed subjects' anonymity, by giving them a random number so there was no way to associate a decision with a name. We were very cautious in minimizing any kind of audience effects. The experiment was conducted with pencil and paper. Subjects could choose among four different languages for their booklet of instructions: Greek, English, Arabic or French^11^. We enforced the usual experimental practice of not allowing for communication among subjects and ensuring anonymity in decision making. Once the session was ready to start, one of the experimenters explained aloud the general instructions of the experiment and answered possible questions. Subjects were told that one game would be chosen randomly by the social worker at the end of the session. Given that inmates are not allowed to receive money directly, we explained to them that their payment would be credited to their personal prison account, which can be used to buy goods inside the prison.

Afterwards, the experiment started and participants were asked to keep silent until the end of the session. In the sessions conducted in 2016 and 2017, we randomized the order in which the PD and TG were presented and played, although we kept the EET always as the third game. The instructions for each game were read in silent by each subject and they could go through the booklet at their own pace. Three experimenters were present in each session in order to answer any question in private and to assist participants. After making all decisions and filling out the questionnaires, participants left the session and received their payment one day later^12^.

Our sample consists of 176 inmates in total. The mean age of inmates is 36.40 years old, they have 4.21 siblings and 1.09 children on average, and 52% of them are married. The mean sentence is 20.06 years and the remaining sentence is 10.46 years on average^13^. Out of the 176 inmates, 71 were recruited in the 2015 sessions and decided only on the CG, and 105 were recruited in 2016–2017 and decided on the TG, PD, and EET. The data collected in 2016 and 2017 (*N* = 105) are also used in Balafoutas et al. (2020), which we already referred to in section Literature review. For this reason, it is important to clarify the commonalities and differences between the two studies. Two key features in Balafoutas et al. (2020) were the administration of a priming intervention for part of the sample in a between-subjects design, as well as the distinction between an in-group and an out-group: inmates played each of the three games (TG, PD, EET) once with another inmate (in-group) and once with someone from outside prison (out-group), in a within-subjects design. The priming intervention consisted of a piece of text that inmates were asked to write, reflecting on the time they had spent in prison and on how it had affected their behavior (see Balafoutas et al., 2020, for more details).

In the present study, we pool the data from the priming and the control condition, since one can reasonably expect this intervention to be orthogonal to the relationship between psychopathy and economic behavior, which is the research question here^14^. Regarding the distinction between an in-group and an out-group, in this study we only use data on decisions affecting an inmate's in-group (i.e., other inmates). This is due to two reasons: first, in the 2015 sessions all inmates interact with their in-group only, and therefore we do not have out-group data for the corruption game. Second, our interest in this study lies in the nature of the relationship between psychopathy and behavior, without the additional dimension of group favoritism or bias.

## Results

We begin this section by presenting (in Table 3) summary statistics for behavior in the four games played by the inmates in our sample. The table reveals strong statistical variation in behavior across participating inmates, thus facilitating the examination of a relationship between behavior and elicited psychopathic traits. Rates of trusting in the TG (*Trust*), cooperating in the PD (*Cooperation*) and taking the bribe-maximizing decision in the CG (*Bribe max*) are all within a 3- to 6 percentage point distance from 50%, while reciprocal choices (*Reciprocity*) are rather frequent at about two-thirds of all choices. In line with most existing studies in experimental economics, behavior in these games is not in line with the Nash equilibrium for selfish subjects. Trust is observed in almost half of the cases, and it is rewarded by second movers in a majority of interactions. Similarly, cooperation rates in the PD lie (at 55%) between the Nash equilibrium of 0% and the social optimum of 100%. In the CG, mean bribes (of 1.75) are between the social optimum of 0 and any of the three pure strategy Nash equilibria, while officials choose the quality-maximizing instead of the bribe-maximizing in just over half of the cases. All of these points toward a considerable degree of pro-social orientation among inmates. Finally, each of the four distributional preference types (*Spiteful, Inequality Averse, Inequality Loving, Altruistic*) accounts for at least 13% and at most 34% of the sample (the exact number of subjects in each type is also shown in Table 3).

**Table 3:** Summary statistics.

	**% / Mean**	***N***	**St. Dv**.
**TG**			
Trust	44.97%	59	0.50
Reciprocity	65.22%	46	0.48
**PD**			
Cooperation	55.24%	105	0.49
**CG**			
Bribe	1.75	48	1.63
Bribe max	47.83%	23	0.51
**EET**			
Spiteful	20%	21/105	0.40
Inequality averse	13.33%	14/105	0.34
Inequality loving	34.28%	36/105	0.48
Altruistic	32.38%	34/105	0.47
**LSRP**			
Primary	27.40	153	12.32
Secondary	17.50	158	7.57
Total	44.58	151	18.65

Turning to an examination of our main research question regarding the relationship between psychopathy and behavior, Table 4 reports mean values of psychopathy as measured in the LSRP, differentiating between primary, secondary, and total psychopathy and linking it to behavior in each of the four games^15^. In particular, LSRP scores are compared across two sub-groups (yes vs. no) of subjects in each game. For each comparison, the table reports *p*-values from two-tailed *t*-tests.

**Table 4:** Social behavior and psychopathy.

	**LSRP Primary**		**LSRP Secondary**		**Total**	
	**Yes**	**No**	**Yes**	**No**	**Yes**	**No**
Trust	32.59 (*N* = 17)	35.13 (*N* = 23)	23.16 (*N* = 19)	22.61 (*N* = 23)	56.88 (*N* = 16)	57.64 (*N* = 22)
	*p = 0.43*		*p = 0.79*		*p = 0.88*	
Reciprocity	33.19 (*N* = 26)	41.15 (*N* = 13)	22.04 (*N* = 28)	23.57 (*N* = 14)	54.64 (*N* = 26)	64.77 (*N* = 13)
	p = 0.02**		*p = 0.34*		p = 0.02**	
Cooperation	33.12 (*N* = 49)	37.9 (*N* = 30)	21.67 (*N* = 49)	24.14 (*N* = 35)	54.68 (*N* = 47)	62.37 (*N* = 30)
	p = 0.04**		p = 0.05**		p = 0.02**	
Spiteful	37.92 (*N* = 12)	34.40 (*N* = 67)	25.53 (*N* = 13)	22.18 (*N* = 71)	64.82 (*N* = 11)	56.48 (*N* = 66)
	*p = 0.26*		p = 0.05**		p = 0.07*	
Altruistic	35.21 (*N =* 14)	34.88 (*N =* 65)	22 (*N =* 13)	22.83 (*N =* 71)	56.92 (*N =* 13)	57.83 (*N =* 64)
	*p = 0.91*		*p = 0.64*		*p = 0.83*	
Inequality Averse	33.14 (*N =* 28)	35.92 (*N =* 51)	22.81 (*N =* 31)	22.64 (*N =* 53)	55.93 (*N =* 28)	58.67 (*N =* 49)
	*p = 0.24*		*p = 0.90*		*p = 0.42*	
Inequality Loving	35.36 (*N =* 25)	34.74 (*N =* 54)	21.55 (*N =* 27)	23.25 (*N =* 57)	56.88 (*N =* 25)	58.06 (*N =* 52)
	*p = 0.80*		*p = 0.21*		*p = 0.74*	
Bribe Max	26.82 (*N =* 11)	15.72 (*N =* 12)	13.55 (*N =* 11)	10.04 (*N =* 12)	40.37 (*N =* 11)	25.75 (*N =* 12)
	p = 0.02**		p = 0.01***		p = 0.01***	
Bribe	> mean	< mean	> mean	< mean	> mean	< mean
	20.02 (*N =* 51)	17.86 (*N =* 23)	11.21 (*N =* 51)	12.47 (*N =* 23)	31.23 (*N =* 51)	30.32 (*N =* 23)
	*p = 0.35*		*p = 0.25*		*p = 0.76*	

*All variables defined in text. p-values correspond to t-tests comparing the two binary categories created within each variable*.

***, **, *indicate p < 0.01, p < 0.05, and p < 0.1, respectively.

The first two rows in Table 4 relate psychopathy to behavior in the trust game. Neither primary nor secondary psychopathy differs significantly between inmates who displayed trusting behavior in this game. Reciprocity, on the other hand, is significantly linked to the LSRP scale: inmates who do not reciprocate trust score higher on primary (and, as a result, on total) psychopathy than those who reciprocate. Similarly, the third row of the table reveals that inmates who take the antisocial action in the prisoner's dilemma (i.e., those who do not cooperate) score significantly higher on both dimensions of psychopathy (primary and secondary) than those who cooperate.

The EET allows us to classify each experimental participant into one of four types of revealed distributional preferences. On aggregate, we find that secondary psychopathy and total psychopathy differ significantly across the four distributional preference types (*p* = 0.03 and *p* = 0.05, respectively; Kruskal-Wallis tests), while the same is not true for primary psychopathy (*p* = 0.16). Turning to each of the four types in isolation, most differences are insignificant. One observation that stands out, however, is that inmates classified as having spiteful preferences have a significantly higher level of LSRP secondary and LSRP total than the other types combined (see row “Spiteful” in Table 4).

In the corruption game, as in the trust game, the sample is split between inmates deciding in the role of “public officials” and inmates deciding in the role of “firms.” We thus report two behavioral outcomes for this game. The main finding with respect to psychopathy is that public officials who take bribe maximizing decisions—i.e., those who behave antisocially by reducing total welfare—have a significantly higher level of LSRP primary, LSRP secondary and total than those who take quality-maximizing decisions. With respect to the decisions of firms, we split our sample of inmates between those who offer a bribe above vs. below the median and compare LSRP levels across the two. We find no significant differences in any of the LSRP dimensions.

Our results thus show that psychopathy, as elicited in the LSRP, significantly correlates with several behavioral measures in the sample of prison inmates who participated in our experiment. Inmates who do not reciprocate, do not cooperate, who are spiteful and who maximize bribe offers have higher levels of psychopathy than their counterparts, ceteris paribus. For these dimensions, a consistent pattern emerges: inmates with higher scores on the psychopathy scales have a higher tendency toward antisocial behavior^16^.

To confirm the robustness of these findings, in Supplementary Material 1 we also report the results of regressions analyses with trust (Supplementary Table 1), reciprocity (Supplementary Table 2), bribe maximizing decisions (Supplementary Table 3), bribe levels and bribe maximizing behavior in the corruption game (Supplementary Tables 4, 5), and belonging to each of the four distributional preference types (Supplementary Tables 6–9) as dependent variables. The main independent variables are *LSRP Primary, LSRP Secondary*, and *LSRP Total*. In addition to parsimonious specifications that include only psychopathy scores, we estimate (in the Probit regressions for trust, reciprocity and cooperation) specifications that control for a number of inmate characteristics available to us through the prison administration and elicited in the post-experimental surveys. These controls are: time served in prison (*time served*) and total sentence (*total sentence*), in months; a dummy variable (*high security*) equal to one for all inmates in the high security prison; the number of other inmates that someone shares a cell with (*cell share*); education level (coded as 0: none; 1: elementary 2: secondary school; 3: high school; 4: university; 5: master); age, a dummy variable equal to one for married inmates, number of children, and number of siblings^17^.

The regression results confirm all main findings obtained so far, both in the parsimonious and in the full specifications. We document a significant relationship between primary psychopathy and reciprocal behavior in the trust game, between both dimensions of psychopathy and cooperation in the prisoner's dilemma, and between both dimensions of psychopathy and bribe maximizing decisions by inmates in the role of public officials in the corruption game. Regarding distributional preference types, Supplementary Table 6 confirms that higher levels of primary and secondary psychopathy are more likely to be encountered among spiteful types (thereby strengthening the non-parametric test results, which were significant only for secondary psychopathy). In addition, the regression analysis in Supplementary Table 7 points toward a further negative association between higher levels of (primary) psychopathy and pro-social behavior, measured by the likelihood of being classified as an inequality averse type.

## Discussion

Psychopathic personality traits are related to lack of empathy and low inhibition, which would be expected to yield antisocial behavior. In this paper, a population of subjects was recruited among the inmates of two Greek prisons. They were asked to reply to the questions of a self-reported psychopathy scale, the LSRP. They were also faced with four incentivized decision-making experimental tasks which are appropriate to study pro-social (or antisocial) behavior. The four tasks involved decisions affecting oneself and others and were chosen to represent different types of interaction. First, a distributional task, the EET, involved binary dictator-type choices among scenarios regarding own and others' rewards. Second, strategic interaction was involved in a simultaneous (prisoner's dilemma) game involving strategic uncertainty on behalf of both players in each subject pair. Third, in a sequential (trust) game, strategic uncertainty was limited to the first mover, while the second player's decision involved no strategic uncertainty. Finally, a more complex, sequential three-player bribery game involved both asymmetric roles and strategic uncertainty. In all these contexts, psychopathy was found to predict antisocial behavior in more or less the expected way, with the exception of active bribers whose psychopathy scores did not predict their bribing behavior. Specifically, higher psychopathy scores relate to lower levels of reciprocity and cooperation, and a higher probability of passive bribery, in the sense of making bribe-maximizing choices.

From a methodological point of view, the robustness of our findings across different economic and game-theoretic experimental tasks can be seen as a confirmation of the validity of the methods used, including the task used for the measurement of subjects' psychopathy, LSRP. Furthermore, the association of psychopathic traits with antisocial behavior is confirmed in a relatively demanding design, in which a broadly used psychometric instrument is shown to reasonably predict behavior in a series of tasks that have the usual abstract framing of context-free decision-making. This framing has interfered in the way others are perceived as (un)trustworthy. Furthermore, the economic decision- making contexts used here have shown further ways of interpreting the difference between primary and secondary psychopathy. The latter is a good predictor of the lack of reciprocity toward people trusting the subject in the first place. Therefore, the results reported here can be seen as an encouraging sign of the benefits from interdisciplinary approaches in order to address the important issue of external and internal validity of the experimental paradigm in both economics and psychology and ultimately document the existence of behavioral spillovers, not only among different economic decision-making tasks, but also across the borders of the two main behavioral sciences.

Regarding the limitations of our study, there are several domains in which our experimental design could be improved or at least complemented by new experiments. First of all, our results come from male prisons. A natural extension would be to check with female institutionalized subjects whether these results are gender-specific. Similarly, a lot could be gained by studying the behavior of inmates in other countries, in order to identify possible prison-specific and country-specific effects. During the experiments we often got the impression that the volunteering inmates accepted to participate in our sessions out of curiosity regarding our “true” objectives. They seemed to hold suspicions regarding our independence from the prison authorities and the anonymity of our protocols. In that sense, highly psychopathic subjects may have adapted their responses in the LSRP test and even their behavior in the experiments in order to project a better self-image in the eyes of the researchers and, supposedly, the prison authority. Future experiments in prison should try to elicit subjects' trust in the researchers' independence and elicit their beliefs on the intentions of the researchers when running similar studies.

A general caveat of experiments with prisoners is that the sample recruited among volunteering inmates will never be comparable with a naturally occurring similar sample extracted from the general population. The span of ages and nationalities contrasts with the unique gender, and the clustering of education on the lowest levels. Relatedly, a limitation of our study is that it documents a relationship between psychopathic traits and inmates' behavior, but it cannot address the question whether psychopathic individuals are more likely to enter prison, or whether psychopathic trends develop inside prison. In general, the extent to which the experience of incarceration shapes behavior is an open and very interesting research question. The small size of the sample is another issue which makes it difficult for the researchers to consider sufficiently many groups in terms of prisoner typologies in order to account for the numerous individual factors which may underlie behavioral differences. Finally, it is difficult -if not impossible- to find a similar, naturally occurring, sample outside the prison to make behavioral comparisons between the inmates and a baseline with non-inmates.

## Data Availability Statement

The raw data supporting the conclusions of this article will be made available by the authors, without undue reservation.

## Ethics Statement

The studies involving human participants were reviewed and approved by Ethics Committee of the School of Agriculture Policy and Development at the University of Reading. The patients/participants provided their written informed consent to participate in this study.

## Author Contributions

NG and EM contributed with funding issues and logistics of the experimental sessions, including obtaining ethics approval and permission to the specific prisons. NG, EM, and TJ-L performed the experiments in the two prisons. LB and TJ-L performed most of the data analysis and produced the manuscript, helped by NG and AG-G. All authors reviewed, edited and approved the final version.

## Conflict of Interest

The authors declare that the research was conducted in the absence of any commercial or financial relationships that could be construed as a potential conflict of interest.

## Publisher's Note

All claims expressed in this article are solely those of the authors and do not necessarily represent those of their affiliated organizations, or those of the publisher, the editors and the reviewers. Any product that may be evaluated in this article, or claim that may be made by its manufacturer, is not guaranteed or endorsed by the publisher.

## Footnotes

^1^ http://www.prisonstudies.org/sites/default/files/resources/downloads/world_prison_population_list_11th_edition_0.pdf

^2^However, pro-social behavior may also relate to selfish inter-temporal cooperation (collusion), or even a subject's risk attitudes. See for instance Sabater-Grande and Georgantzis (2002).

^3^Gillespie et al. (2013) and Spitzer et al. (2007) consider the connection between psychopathy and ultimatum games.

^4^Regarding the role of gender, we note the existence of strong evidence that psychopathic personality traits as egocentrism, manipulativeness, etc. manifest quite differently among the genders (Brinkley et al., 2008; Croson and Gneezy, 2009).

^5^For instance, Vaughn et al. (2009) demonstrate that young offenders who have been identified with strong expressions of the secondary subtype were more likely to have experienced trauma and abuses in their past, thus supporting the assumption that secondary psychopathy is possibly caused by environmental factors.

^6^Sessions were conducted in different years and although the LSRP test was filled in all sessions, not all four games were applied for all the sample. See subsection 3.3 for details on this aspect of our experimental procedures.

^7^We implemented the strategy method for collecting data on receivers' choices, which means that they were asked to make a choice between the two possible allocations for the event that the sender they were matched with decided to trust them.

^8^It should be noticed that pro-social choices by senders and receivers in the trust game can arise from several motivations, the identification of which is beyond the scope of this work (Cox, 2004; Isoni and Sugden, 2019). The literature commonly refers to such choices as trust (in the case of senders) and reciprocity or trustworthiness (in the case of receivers).

^9^For more details on the classification of types, see Kerschbamer (2015). Note that selfish subjects are a subset of the four other categories and that including them as a separate category does not affect any of our findings.

^10^The Likert-scale items are phrased so as to minimize indication of disapproval for item endorsement.

^11^Instructions in languages other than English were translated from English by native speakers. The experimental instructions can be found in Supplementary Material 2.

^12^We note that there was no attrition during a session: all participants completed all parts of the experiment and none left a session before doing so. However, some participants did not fill out all information in the questionnaires, including a few who did not answer all questions in the LSRP, leading to a smaller effective sample size used in the data analysis.

^13^For more information on the sociodemographic characteristics of the inmates recruited during the sessions in 2016 and 2017 please refer to Balafoutas et al. (2020).

^14^This orthogonality assumption is something that we can test: for all regressions presented in the results section (see in particular Supplementary Tables 1–9 in the Supplementary Material), we have estimated versions in which we add a dummy variable equal to 1 for all inmates in the priming group, as well as interactions between this variable and the LSRP scores. All these terms are insignificant, supporting the validity of pooling the data from the two groups in the analysis. These regressions are not shown in the paper in the interest of brevity but they are available upon request.

^15^We perform a Cronbach's alpha test with LSRP primary, secondary and total leading to a scale reliability coefficient of 0.87.

^16^Given the framing in the CG, an alternative interpretation of the antisocial behavior of inmates is that it reflects their beliefs about the prison personnel that they consider a public official. For instance, if they believe guards are corrupt, they are more likely to engage in bribe-maximizing behavior to express how they believe guards tend to act. In this case egocentric inmates may exhibit this antisocial behavior to show their discomfort in their relationship with guards.

^17^In the corruption game these control variables are available only for a small sub-sample of inmates, thus not yielding enough degrees of freedom to estimate the full specifications.
