# Supplementary material for: Psychopathy and Economic Behavior Among Prison Inmates: An Experiment
Source: Front Psychol. 2021 Sep 20;12:732184. doi: 10.3389/fpsyg.2021.732184 (PMC8488147; doi:10.3389/fpsyg.2021.732184)
Supplement: Supplementary file 1 [file Data_Sheet_1.PDF]

Supplementary Material

A.1. Regression Analysis

In this section we present an econometric data analysis that links inmates' behavior in the different games to their elicited psychopathy levels.

Table A1. Regression analysis on Trust and LSRP

	(1)	(2)	(3)	(4)	(5)	(6)
LSRP Total	-0.002			0.033		
	(0.014)			(0.030)		
LSRP Primary		-0.017			0.020	
		(0.021)			(0.038)	
LSRP Secondary			0.008			-0.030
			(0.030)			(0.044)
time served				-0.053	-0.076	-0.072
				(0.106)	(0.100)	(0.070)
total sentence				0.005	-0.007	-0.010
				(0.019)	(0.018)	(0.019)
high security				0.752	0.979	0.358
				(0.978)	(0.970)	(0.921)
married				0.215	-0.282	0.177
				(0.788)	(0.731)	(0.616)
cell share				-0.861**	-0.590**	-0.603**
				(0.428)	(0.284)	(0.271)
age				0.122*	0.043	0.034
				(0.063)	(0.038)	(0.040)
education				0.180	0.325	-0.122
				(0.376)	(0.345)	(0.293)
# children				-0.367	-0.111	0.152
				(0.396)	(0.343)	(0.185)
# siblings				0.016	0.037	0.024
				(0.057)	(0.053)	(0.047)
N	38	40	42	31	33	35

Notes: Dependent variable: *Trust*, equal to 1 if a subject-sender chose to trust in the TG, and 0 otherwise. All specifications are estimated with Probit models. Standard errors in parantheses. Independent variables as defined in the text. N varies across specifications depending on the number of available observations. *, ** indicates statistical significance at the 10%, 5% level, respectively.

Table A2. Regression analysis on Reciprocity and LSRP

	(1)	(2)	(3)	(4)	(5)	(6)
LSRP Total	-0.039**			-0.044*		
	(0.017)			(0.024)		
LSRP Primary		-0.052**			-0.055*	
		(0.023)			(0.029)	
LSRP Secondary			-0.043			-0.077
			(0.043)			(0.072)
time served				0.030	0.018	0.060
				(0.081)	(0.083)	(0.077)
total sentence				0.014	0.015	0.011
				(0.034)	(0.033)	(0.033)
high security				-0.769	-0.721	-0.917
				(1.360)	(1.338)	(1.323)
married				-1.081	-0.960	-1.459*
				(0.977)	(0.952)	(0.862)
cell share				-0.026	-0.016	-0.029
				(0.180)	(0.180)	(0.174)
age				-0.078**	-0.077**	-0.080**
				(0.036)	(0.036)	(0.035)
education				-0.007	-0.032	0.012
				(0.335)	(0.330)	(0.325)
# children				0.287	0.274	0.443
				(0.389)	(0.381)	(0.360)
# siblings				-0.014	-0.031	0.003
				(0.079)	(0.079)	(0.077)
N	39	39	42	36	36	38

Notes: Dependent variable: *Reciprocity*, equal to 1 if a subject-receiver chose to reciprocate in the TG by sharing the total amount with the sender, and 0 otherwise. All specifications are estimated with Probit models. Standard errors in parantheses. Independent variables as defined in the text. N varies across specifications depending on the number of available observations. *, ** indicates statistical significance at the 10%, 5% level, respectively.

Table A3. Regression analysis on Cooperation and LSRP

	(1)	(2)	(3)	(4)	(5)	(6)
LSRP Total	-0.025**			-0.035***		
	(0.012)			(0.014)		
LSRP Primary		-0.031**			-0.042**	
		(0.015)			(0.019)	
LSRP Secondary			-0.048*			-0.058*
			(0.025)			(0.030)
time served				0.026	0.011	0.0217
				(0.055)	(0.054)	(0.045)
total sentence				-0.033**	-0.027*	-0.022
				(0.015)	(0.015)	(0.014)
high security				1.061	0.769	0.911
				(0.788)	(0.758)	(0.714)
married				-0.087	0.114	0.020
				(0.500)	(0.480)	(0.378)
cell share				-0.275**	-0.274**	-0.244**
				(0.123)	(0.119)	(0.117)
age				-0.040*	-0.027	-0.023
				(0.024)	(0.022)	(0.021)
education				-0.054	-0.071	-0.083
				(0.200)	(0.195)	(0.186)
children				0.296	0.194	0.045
				(0.201)	(0.188)	(0.121)
siblings				-0.082*	-0.090*	-0.062
				(0.050)	(0.050)	(0.045)
N	77	79	84	67	69	73

Notes: Dependent variable: *Cooperation*, equal to 1 if a subject chose to cooperate in the PD, and 0 if he chose to defect. All specifications are estimated with Probit models. Standard errors in parantheses. Independent variables as defined in the text. N varies across specifications depending on the number of available observations. *, **, *** indicates statistical significance at the 10%, 5%, 1% level, respectively.

Table A4. Regression analysis on Bribe-Maximizing behavior and LSRP

	(1)	(2)	(3)
LSRP Total	0.061**		
	(0.025)		
LSRP Primary		0.066**	
		(0.029)	
LSRP Secondary			0.274**
			(0.114)
N	23	23	23

Notes: Dependent variable: *Bribe max*, equal to 1 if a subject chose the bribe-maximizing option in the role of public official in the CG, and 0 otherwise. All specifications are estimated with Probit models. Standard errors in parantheses. ** indicates statistical significance at the 5% level.

Table A5. OLS regression on Bribe

	(1)	(2)	(3)
LSRP Total	0.020		
	(0.023)		
LSRP Primary		0.039	
		(0.031)	
LSRP Secondary			-0.010
			(0.052)
N	48	48	48

Notes: Dependent variable: *Bribe*, offered by subjects in the role of firms in the CG. All specifications are estimated with Ordinary Least Squares. Standard errors in parantheses.

Table A6. Regression analysis on Spiteful types and LSRP

	(1)	(2)	(3)	(4)	(5)	(6)
LSRP Total	0.022*					0.091**
	(0.012)					(0.036)
LSRP Primary		0.019		0.090**		
		(0.017)		(0.035)		
LSRP Secondary			0.061*		0.210**	
			(0.031)		(0.091)	
time served				0.153*	0.145**	0.197**
				(0.080)	(0.062)	(0.096)
total sentence				0.003	0.009	0.010
				(0.017)	(0.018)	(0.018)
high security				0.682	0.461	0.663
				(1.039)	(1.042)	(1.083)
married				0.307	0.447	0.543
				(0.776)	(0.795)	(0.852)
cell share				-0.250	-0.157	-0.186
				(0.232)	(0.215)	(0.242)
age				-0.065	-0.016	-0.066
				(0.044)	(0.040)	(0.047)
education				1.124***	1.236**	1.378**
				(0.436)	(0.484)	(0.565)
# children				-0.128	-0.265	-0.148
				(0.352)	(0.366)	(0.390)
# siblings				0.059	0.006	0.066
				(0.049)	(0.041)	(0.050)
N	77	79	84	69	73	67

Notes: Dependent variable: *Spiteful*, equal to 1 if a subject is classified as a spiteful type in the EET, and 0 otherwise. All specifications are estimated with Probit models. Standard errors in parantheses. Independent variables as defined in the text. N varies across specifications depending on the number of available observations. *, **, *** indicates statistical significance at the 10%, 5%, 1% level, respectively.

Table A7. Regression analysis on Inequality Averse types and LSRP

	(1)	(2)	(3)	(4)	(5)	(6)
LSRP Total	-0.009					-0.034**
	(0.011)					(0.015)
LSRP Primary		-0.018		-0.057***		
		(0.015)		(0.022)		
LSRP Secondary			0.003		-0.025	
			(0.024)		(0.032)	
time served				-0.167**	-0.136***	-0.150**
				(0.066)	(0.053)	(0.061)
total sentence				-0.037*	-0.0173	-0.034*
				(0.021)	(0.016)	(0.029)
high security				1.427	0.591	1.232
				(0.909)	(0.744)	(0.894)
married				-1.324**	-0.296	-1.130*
				(0.641)	(0.416)	(0.632)
cell share				-0.180	-0.163	-0.207
				(0.126)	(0.120)	(0.126)
age				-0.042	-0.010	-0.034
				(0.026)	(0.023)	(0.026)
education				-0.458**	-0.399*	-0.420*
				(0.232)	(0.209)	(0.226)
# children				0.559**	0.089	0.477**
				(0.239)	(0.133)	(0.236)
# siblings				-0.179**	-0.168**	-0.184**
				(0.088)	(0.077)	(0.089)
N	77	79	84	69	73	67

Notes: Dependent variable: *Inequality averse*, equal to 1 if a subject is classified as an inequality averse type in the EET, and 0 otherwise. All specifications are estimated with Probit models. Standard errors in parantheses. Independent variables as defined in the text. N varies across specifications depending on the number of available observations. *, **, *** indicates statistical significance at the 10%, 5%, 1% level, respectively.

Table A8. Regression analysis on Inequality Loving types and LSRP

	(1)	(2)	(3)	(4)	(5)	(6)
LSRP Total	-0.004					0.009
	(0.011)					(0.014)
LSRP Primary		0.004		0.019		
		(0.015)		(0.020)		
LSRP Secondary			-0.030		-0.024	
			(0.025)		(0.032)	
time served				0.055	0.020	0.034
				(0.053)	(0.045)	(0.053)
total sentence				0.0218	0.018	0.027*
				(0.0143)	(0.015)	(0.015)
high security				-1.603	-1.649	-1.993*
				(0.990)	(1.090)	(1.051)
married				0.991*	0.432	1.198**
				(0.518)	(0.405)	(0.551)
cell share				0.066	0.022	0.041
				(0.120)	(0.121)	(0.123)
age				0.033	0.025	0.044*
				(0.022)	(0.022)	(0.024)
education				0.0843	-0.050	0.008
				(0.203)	(0.193)	(0.210)
# children				-0.265	-0.026	-0.350
				(0.200)	(0.131)	(0.216)
# siblings				0.067	0.069	0.059
				(0.045)	(0.045)	(0.044)
N	77	79	84	69	73	67

Notes: Dependent variable: *Inequality loving*, equal to 1 if a subject is classified as an inequality loving type in the EET, and 0 otherwise. All specifications are estimated with Probit models. Standard errors in parantheses. Independent variables as defined in the text. N varies across specifications depending on the number of available observations. *, ** indicates statistical significance at the 10%, 5% level, respectively.

Table A9. Regression analysis on Altruistic types and LSRP

	(1)	(2)	(3)	(4)	(5)	(6)
LSRP Total	-0.003					-0.007
	(0.012)					(0.016)
LSRP Primary		0.002		-0.006		
		(0.017)		(0.021)		
LSRP Secondary			-0.015		-0.015	
			(0.030)		(0.040)	
time served				-0.052	-0.012	-0.016
				(0.070)	(0.0638)	(0.070)
total sentence				0.017	0.010	0.009
				(0.016)	(0.016)	(0.016)
high security				-0.132	0.346	0.292
				(0.745)	(0.804)	(0.807)
married				0.028	-0.378	-0.341
				(0.546)	(0.538)	(0.596)
cell share				0.315^{**}	0.363^{**}	0.355^{**}
				(0.147)	(0.152)	(0.151)
age				0.023	-0.001	-0.003
				(0.028)	(0.030)	(0.031)
education				-0.293	-0.148	-0.159
				(0.246)	(0.253)	(0.257)
# children				-0.344	-0.191	-0.168
				(0.262)	(0.230)	(0.275)
# siblings				-0.040	-0.022	-0.030
				(0.077)	(0.069)	(0.073)
N	77	79	84	69	73	67

Notes: Dependent variable: *Altruistic*, equal to 1 if a subject is classified as an altruistic type in the EET, and 0 otherwise. All specifications are estimated with Probit models. Standard errors in parantheses. Independent variables as defined in the text. N varies across specifications depending on the number of available observations. ** indicates statistical significance at the 5% level.

A.2. Sample experimental instructions

Trust Game, first mover

You are PLAYER 1. You have to decide on which strategy you prefer. You can choose between:

- strategy 1 and the game continues (in which case player 2 decides) or
- strategy 2 and then the game ends

-If YOU choose strategy 1 the money you will earn will depend on the strategy that PLAYER 2 will choose:

-If PLAYER 2 chooses strategy A, you both will earn 20 euros each.

- If PLAYER 2 chooses strategy B, you will earn 5 euros and your partner will earn 35 euros.

-If YOU choose Strategy 2, the game ends with earnings of 10 euros each.

Notice that at the moment of deciding, you will not know the option your partner will choose, but you know that your partner is someone in prison.

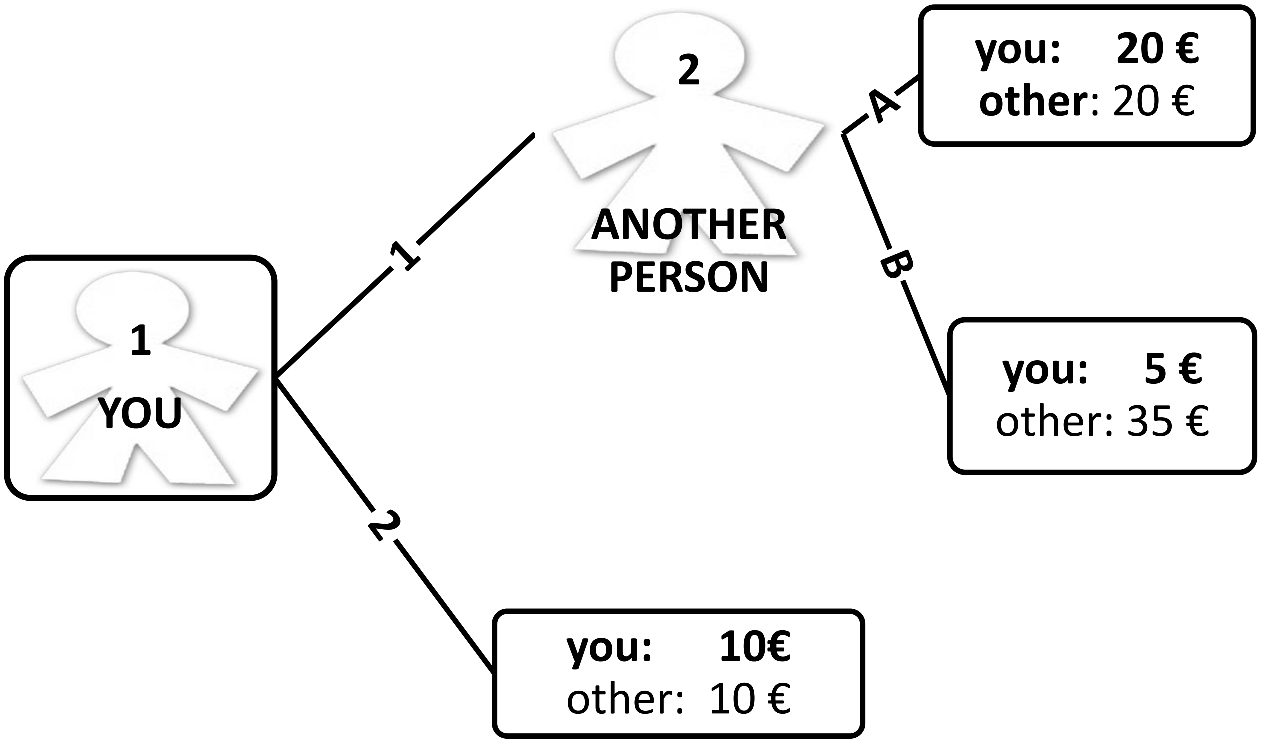

Trust Game, second mover

You are PLAYER 2. You have to decide on which strategy you prefer.

Before you, we asked to another person who is in the prison, to play as PLAYER 1. The other person decided to play Strategy 1 so now is YOUR turn to choose.

-If YOU choose strategy A you will earn 20 euros each one.

-If YOU choose strategy B you will earn 35 euros and he will earn 5 euros.

Notice that the other person is someone in the prison.

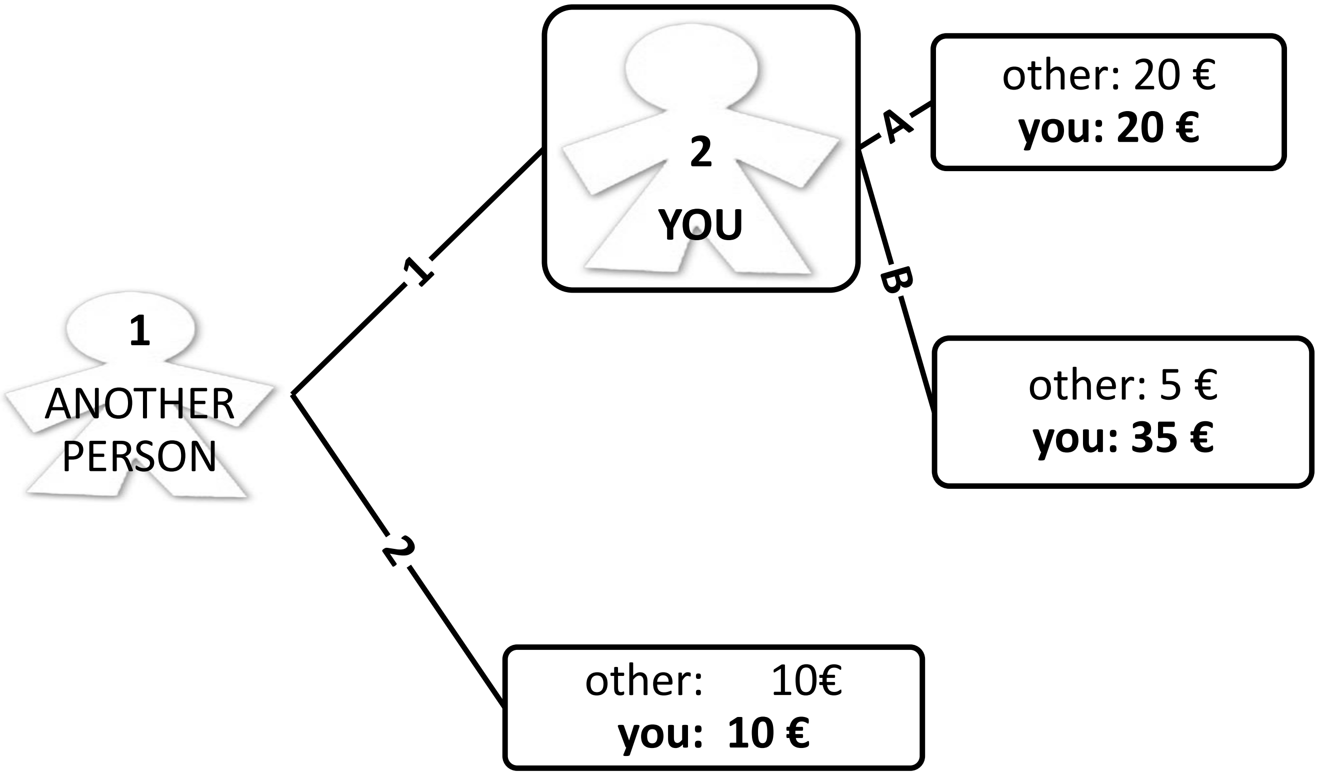

Prisoner's Dilemma

You (and other person) have to decide which option you prefer: A or B.

You don't know the option chosen by the other person, but you know that the other person is someone in the prison.

Therefore,

-if you choose A and the other person chooses A, you both will earn 7 euros

-if you choose B and the other person chooses B, you both will earn 3 euros

-if you choose A and the other person chooses B, you will earn 1 euro and the other person will earn 9 euros.

-if you choose B and the other person chooses A, you will earn 9 euros and the other person will earn 1 euro.

		Another person inside prison	
		**A**	**B**
**YOU**	**A**	**You: 7 €**<br>Other person: 7 €	**You: 1 €**<br>Other person: 9 €
	**B**	**You: 9 €**<br>Other person: 1 €	**You: 3 €**<br>Other person: 3 €

Equality Equivalence Test

You have to decide on how you would like to distribute an amount of money between YOU and another person who is in the prison.

For each row please choose whether you prefer the distribution on the LEFT or the distribution on the RIGHT. Please, do not forget to make a choice for each row.

For example: take a look at the first row. If you choose the distribution on the RIGHT it means that you prefer to get 4 euros for you and 4 euros for the other person than 3.20 euros for you and 5.20 euros for the other person.

You get	Another person inside the prison gets		You get	Another person inside the prison gets

LEFT			RIGHT	
3.2 €	5.2 €		4 €	4 €
3.6 €	5.2 €		4 €	4 €
4 €	5.2 €		4 €	4 €
4.4 €	5.2 €		4 €	4 €
4.8 €	5.2 €		4 €	4 €

LEFT			RIGHT	
3.2 €	2.8 €		4 €	4 €
3.6 €	2.8 €		4 €	4 €

4 €	2.8 €		4 €	4 €
4.4 €	2.8 €		4 €	4 €
4.8 €	2.8 €		4 €	4 €

Corruption Game

You will be assigned one of two roles: `a firm' or `an official'. Your role is randomly assigned. You will be anonymously and randomly assigned to a group of three players: two firms and an official.

Decision Making

You receive an endowment of 10 ExCU.

If you are a firm: You compete with the other firm of your group for the license of a public project, the quality of which, is beneficial to all players in the group. You have to post bids on the quality of your project and a monetary transfer which you wish to send privately to the official in your group if you are chosen to undertake the project. Firms' bids are made simultaneously, so that each firm can only know its own bids, but not the bids of the other firm. The quality and the transfer to the official must sum 10, so that if your quality bid is 9 your transfer to the official in case you win will be 1. If you win the auction, apart from your endowment, you earn an extra profit. In that case, you also have to spend on the transfer to the official double the amount you promised in your bid. If you lose the auction, apart from your initial endowment, your earnings include a profit which is proportional to the quality of the winning project.

If you are an official: You receive the bids from the firms in your group. Then, you have to choose one of the two projects. Apart from your initial endowment, your earnings include a profit which is proportional to the winner's quality plus the amount, if any, privately transferred to you by the winner.

Exact calculation of profits

From the description of strategies and earnings above, the specific formulas used to calculate your profits (π) are a function of the quality (Q) and transfer (B) bids of the winner, as shown below:

1) $\pi_{official} = 10 + \frac{1}{2}.Q_{winner} + B_{winner}$
2) $\pi_{winner} = 10 + \frac{1}{2}.Q_{winner} - 2\,.B_{winner} + 10$
3) $\pi_{loser} = 10 + \frac{1}{2}.Q_{winner}$

Information received

If you are a firm: You will receive information on which firm won the license, as well as your profit.

If you are an official: After firms have made their decisions, their quality and transfer bids will be shown to you before you make a decision. Once you select the winning firm, you will receive information on your profits.

Monetary rewards

The amount of money you will earn from this part of the experiment will be equal to your profits in multiplied by an equivalence ratio of 1 ExCU = ½ Euro.

A.3. Levenson Self-Report Psychopathy (LSRP) scale

**1. Success is based on survival of the fittest; I am not concerned about the losers.**					
**DISAGREE**	**1**	**2**	**3**	**4**	**AGREE**
**2. I find myself in the same kinds of trouble, time after time.**					
**DISAGREE**	**1**	**2**	**3**	**4**	**AGREE**
**3. For me, what's right is whatever I can get away with.**					
**DISAGREE**	**1**	**2**	**3**	**4**	**AGREE**
**4. I am often bored.**					
**DISAGREE**	**1**	**2**	**3**	**4**	**AGREE**
**5. In today's world, I feel justified in doing anything I can get away with to succeed.**					
**DISAGREE**	**1**	**2**	**3**	**4**	**AGREE**
**6. I find that I am able to pursue one goal for a long time.**					
**DISAGREE**	**1**	**2**	**3**	**4**	**AGREE**
**7. My main purpose in life is getting as many goodies as I can.**					
**DISAGREE**	**1**	**2**	**3**	**4**	**AGREE**
**8. I don't plan anything very far in advance.**					
**DISAGREE**	**1**	**2**	**3**	**4**	**AGREE**
**9. Making a lot of money is my most important goal.**					
**DISAGREE**	**1**	**2**	**3**	**4**	**AGREE**
**10. I quickly lose interest in tasks I start.**					
**DISAGREE**	**1**	**2**	**3**	**4**	**AGREE**

**11. I let others worry about higher values; my main concern is with the bottom line**					
**DISAGREE**	**1**	**2**	**3**	**4**	**AGREE**
**12 Most of my problems are due to the fact that other people just don't understand me.**					
**DISAGREE**	**1**	**2**	**3**	**4**	**AGREE**
**13. People who are stupid enough to get ripped off usually deserve it.**					
**DISAGREE**	**1**	**2**	**3**	**4**	**AGREE**
**14. Before I do anything, I carefully consider the possible consequences.**					
**DISAGREE**	**1**	**2**	**3**	**4**	**AGREE**
**15. Looking out for myself is my top priority.**					
**DISAGREE**	**1**	**2**	**3**	**4**	**AGREE**
**16 I have been in a lot of shouting matches with other people.**					
**DISAGREE**	**1**	**2**	**3**	**4**	**AGREE**
**17. I tell other people what they want to hear so that they will do what I want them to do.**					
**DISAGREE**	**1**	**2**	**3**	**4**	**AGREE**
**18. When I get frustrated, I often "let off steam" by blowing my top.**					
**DISAGREE**	**1**	**2**	**3**	**4**	**AGREE**
**19. I would be upset if my success came at someone else's expense.**					
**DISAGREE**	**1**	**2**	**3**	**4**	**AGREE**
**20. Love is overrated.**					
**DISAGREE**	**1**	**2**	**3**	**4**	**AGREE**
**21. I often admire a really clever scam.**					

**DISAGREE**	**1**	**2**	**3**	**4**	**AGREE**
**22. I make a point of trying not to hurt others in pursuit of my goals.**					
**DISAGREE**	**1**	**2**	**3**	**4**	**AGREE**
**23. I enjoy manipulating other people's feelings.**					
**DISAGREE**	**1**	**2**	**3**	**4**	**AGREE**
**24. I feel bad if my words or actions cause someone else to feel emotional pain.**					
**DISAGREE**	**1**	**2**	**3**	**4**	**AGREE**
**25. Even if I were trying very hard to sell something, I wouldn't lie about it.**					
**DISAGREE**	**1**	**2**	**3**	**4**	**AGREE**
**26. Cheating is not justified because it is unfair to others.**					
**DISAGREE**	**1**	**2**	**3**	**4**	**AGREE**
